# Postoperative atrial fibrillation and atrial epicardial fat: Is there a link?

**DOI:** 10.1016/j.ijcha.2022.100976

**Published:** 2022-02-21

**Authors:** Claudia A.J. van der Heijden, Sander Verheule, Jules R. Olsthoorn, Casper Mihl, Lexan Poulina, Sander M.J. van Kuijk, Samuel Heuts, Jos G. Maessen, Elham Bidar, Bart Maesen

**Affiliations:** a Department of Cardiothoracic Surgery, Maastricht University Medical Centre, Maastricht, the Netherlands; b Department of Physiology, Maastricht University, Maastricht, the Netherlands; c Cardiovascular Research Institute Maastricht (CARIM), Maastricht, the Netherlands; d Department of Cardiothoracic Surgery, Catharina Hospital Eindhoven, Eindhoven, the Netherlands; e Department of Radiology and Nuclear Medicine, Maastricht University Medical Centre, Maastricht, the Netherlands; f Department of Clinical Epidemiology and Medical Technology Assessment, Maastricht University Medical Centre, Maastricht, the Netherlands

**Keywords:** Postoperative atrial fibrillation, Epicardial adipose tissue, Computed tomography scan, Cardiac surgery, AF, Atrial Fibrillation, AV, Aortic Valve, BMI, Body Mass Index, BSA, Body Surface Area, CABG, Coronary Artery Bypass Graft, CCTA, Coronary Computed Tomographic Angiography, CM, Contrast Management, CT, Computed Tomography, EAT, Epicardial Adipose Tissue, EAT-V, EAT-Volume, HU, Hounsfield Units, kV, Kilovoltage, LA, Left Atrial, LAVI, LA Volume Index, LVEF, Left Ventricular Ejection Fraction, ml, Millilitre, MYO, Myocardial, MYO-V, MYO-Volume, POAF, Post-Operative AF, ROI, Regions Of Interest, SR, Sinus Rhythm

## Abstract

**Background:**

Atrial Epicardial Adipose Tissue (EAT) is presumably involved in the pathogenesis of atrial fibrillation (AF). The transient nature of postoperative AF (POAF) suggests that surgery-induced triggers provoke an unmasking of a pre-existent AF substrate. The aim is to investigate the association between the volume of EAT and the occurrence of POAF. We hypothesise that the likelihood of developing POAF is higher in patients with high compared to low left atrial (LA) EAT volumes.

**Methods:**

Quantification of LA EAT based on the Hounsfield Units using custom made software was performed on pre-operative coronary computed tomography angiography scans of patients who underwent cardiac surgery between 2009 and 2019. Patients with mitral valve disease were excluded.

**Results:**

A total of 83 patients were included in this study (CABG = 34, aortic valve = 33, aorta ascendens n = 7, combination n = 9), of which 43 patients developed POAF. The EAT percentage in the LA wall nor indexed EAT volumes differed between patients with POAF and with sinus rhythm (all P > 0.05). In multivariable analysis, age and LA volume index (LAVI) were the only independent predictors for early POAF (OR: 1.076 and 1.056, respectively).

**Conclusions:**

As expected, advanced age and LAVI were independent predictors of POAF. However, the amount of local EAT was not associated with the occurrence of AF after cardiac surgery. This suggests that the role of EAT in POAF is rather limited, or that the association of EAT in the early phase of POAF is obscured by the dominance of surgical-induced triggers.

## Highlights

• Detailed local EAT analysis does not differ between POAF and non-POAF.
• General rather than local effects of EAT play a role in the onset of early POAF.
• The dominance of surgical induced factors may obscure the potential role of EAT.

## 1 Introduction

The onset of atrial fibrillation (AF) after cardiac surgery is associated with an increased risk of stroke, higher hospitalization costs and decreased short- and long-term survival [1], [2]. In addition, the occurrence of postoperative AF (POAF), although often considered to be a transient phenomenon, is a predictor of later recurrences of AF [3]. For example, patients with a single episode of early POAF have an eightfold increased risk in developing AF later on [2]. Although the exact pathophysiological mechanisms underlying this arrhythmia are still incompletely understood, it is believed that POAF is the result of an interplay between acute factors related to the early postoperative period, and pre-existent substrate related to e.g. heart disease and ageing of the heart [4].

Epicardial adipose tissue (EAT) [5], defined as the volume of adipose tissue between the myocardium and the visceral pericardium, can lead to structural remodelling and AF substrate development [6]. However, the association between EAT and AF after cardiac surgery remains unclear. Given the transient character of early postoperative AF (POAF), and the role of EAT early in the development of the AF substrate, it is interesting to evaluate the role of EAT in the occurrence of AF after cardiac surgery. Therefore, this study aims to investigate the association between EAT and early POAF in patients undergoing cardiac surgery. We hypothesize that patients with higher EAT volumes are more likely to develop POAF after cardiac surgery than patients with low EAT volumes.

## 2 Patients and methods

### 2.1 Study design and setting

This retrospective study was approved by the Institutional Review Board (IRB) and Ethics Committee (METC 2018–0448), and analysed anonymously in accordance with IRB guidelines. The study complies with the ethical principles of the Helsinki Declaration.

Consecutive patients undergoing coronary artery bypass graft (CABG), aorta ascendens surgery or surgical aortic valve replacement (AVR) at the Maastricht University Medical Centre+ (the Netherlands) from the 1st of November 2009 until the 31st of December 2019 were potential candidates. We included patients who had undergone a preoperative coronary computed tomographic angiography (CCTA) prior to the surgical procedure. Patients with a history of AF were included in the study to compare baseline characteristics with non-AF patients, but excluded from the primary analysis comparing EAT between POAF and SR to reduce confounding.

### 2.2 Data selection

POAF was defined as an (a)symptomatic episode of supraventricular arrhythmia of at least 30 s detected by continuous rhythm monitoring or a 12-lead ECG [7]. Postoperative continuous monitoring was used at least three and four days in patients who underwent CABG or aortic (valve) surgery respectively. In the case of symptoms such as palpitations a 12-lead ECG was obtained.

### 2.3 Computed tomographic imaging protocol

All CT scans were conducted using a standardized protocol, using a second generation dual source CT scanner (Somatom Definition Flash, Siemens Medical Solutions, Erlangen, Germany; slice collimation 128x0.6 mm) or a third generation dual source CT-scanner (Somatom Force, Siemens Healthineers, Forchheim, Germany; slice collimation 192x0.6 mm). Tube voltage of scan protocols varied between 80 and 120 kV. The contrast management (CM) injection protocol consisted of 300 mg l/ml Iopromide (Ultravist, Bayer Healthcare, Berlin, Germany), prewarmed to body temperature (37 ˚C; 99 ˚F), injected using a dual-head CT power injector (Stellant, Bayer).

Depending on the heart rate, a different scan protocol was used. In patients with a stable heart rate < 60 bpm, a prospectively ECG-triggered “high pitch” spiral protocol was used (“Flash”-technique). A prospectively triggered “adaptive sequence” protocol (prospective sequential data acquisition) was used in patients with a stable heart rate between 60 and 90 bpm. A retrospectively gated helical protocol was used in patients with an irregular heart rhythm or with a heart rate ≥ 90 bpm. To reduce confounding factors in the analysis, all patients who were scanned with an older generation CT-scanner (Brilliance 64, Philips, Healthcare, Best, Netherlands) were excluded, as differences in scan and CM injection parameters (e.g. tube voltage [kV], total injected CM volume, flow rate) have an effect on the attenuation of EAT as measured by Hounsfield Units (HU).

### 2.4 Atrial delineation

**Fig. 1:**
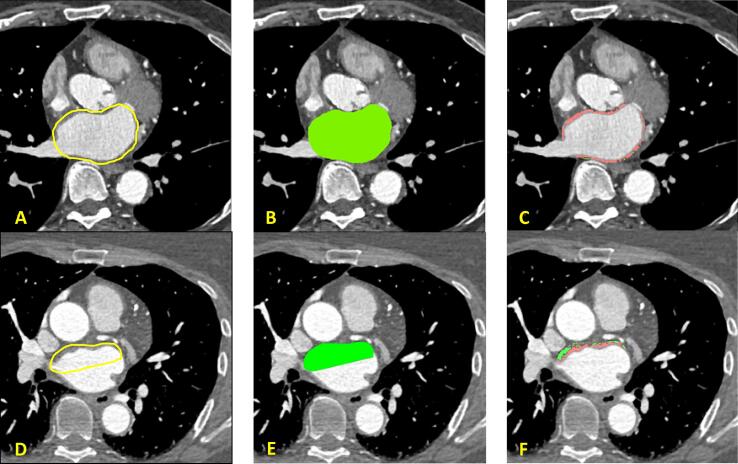
Coronary computed tomography angiography images with LA EAT and MYO measurements in the whole wall (A–C) and the roof specifically (D–F). (A and D) Manual delineation of the LA (yellow line). (B and E) Selection of the region of interest (green colour). (C and F) Pixels containing MYO (red colour) and EAT (green colour) based on HU ranging from 10 to 120 HU and −30 to −190 HU respectively. EAT = epicardial adipose tissue; MYO = myocardial tissue; LA = left atrium; HU = Hounsfield unit.

LA EAT was manually delineated using an image processing program designed for scientific multidimensional images (ImageJ software, U.S. National Institutes of Health, Bethesda, MD, USA) and a pen tablet (Wacom, Kazo, Saitama, Japan) [8]. The regions of interest (ROI) were manually delineated, starting at the basal slice above the mitral valve annulus. In addition to this the left atrial appendage was delineated [8]. Delineation of LA EAT was performed by an independent assessor (C.A.J. van der H.) blinded for patient characteristics and rhythm outcome. In the case of inconclusive quantification, scans were reviewed by a second assessor (B.M.) (Fig. 1A and D).

### 2.5 Epicardial adipose tissue calculation

Custom-made semi-automated software developed in ImageJ (AdipoQuant, developed by S.V.) was used to quantify the number of pixels of EAT and myocardial tissue (MYO) based on the HU per ROI. A range within −30 to −190 HU was defined as EAT, while MYO was defined as pixels ranging from 10 to 120 HU (Fig. 1B-C and E-F) [8], [9]. Thereafter, total LA EAT and MYO volumes (EAT-V and MYO-V) in millilitre (ml) were calculated using several parameters obtained from the metadata of the CCTA, including pixel size, slice thickness and the spacing between slices. EAT-V and MYO-V were used to calculate the percentage of EAT in the LA myocardial wall (EAT-V/[EAT-V + MYO-V]*100). Consequently, EAT-V and EAT-V indexed to LA-volume (ml) (EAT-LA) were indexed to several clinical parameters that potentially influence the volume of EAT, including body surface area (BSA), body mass index (BMI), MYO-V and left atrial dimension. To determine whether EAT location differed within and between groups, a separate EAT-V analysis at the LA roof was performed. Subsequently, roof EAT-V and non-roof EAT-V (total EAT-V minus roof-EAT-V) were compared (Fig. 1D–F).

### 2.6 Statistical analysis

Statistical analysis was performed based on rhythm outcome (POAF versus SR) using SPSS version 25.0 (SPSS Inc., Chicago, IL, USA). Normality of distribution of continuous variables was tested using the Shapiro-Wilk test and by visual inspection of histograms. A student’s *t*-test was performed for continuous means in case of normality (mean ± standard deviation) and a Mann-Whitney-*U* test in case of non-normally distributed data (median and interquartile range). A Pearson’s χ^2^ test or Fisher’s exact test was performed to compare categorical variables between both groups. Univariate analyses were performed using Pearson’s r or Spearman’s Rho, depending on data distribution, between clinical characteristics and EAT variables. Finally, multivariable regression analysis was performed to identify independent predictors of POAF. For all analyses, differences were considered statistically significant if *P* < 0.05.

## 3 Results

### 3.1 Study population

Of the screened population, 107 patients that underwent cardiac surgery at Maastricht University Medical Centre between 2009 and 2019 had a preoperative CCTA. Eleven patients were excluded due to incomplete or suboptimal quality of the images and 13 patients were excluded due to being scanned with an older scanner, as outlined in the methods. In addition, 12 patients had a history of AF and were therefore excluded from the EAT analysis only. In total, 83 patients were retrospectively included in our analysis.

Patients were on average 61 ± 10 years old, 24% was female and 42% underwent stand-alone CABG surgery. Patients with POAF had a greater LA volume (82 ± 28 ml vs. 68 ± 21 ml, *P* = 0.039) and LA volume index (LAVI) (41 ± 13 vs. 35 ± 10 ml/m^2^, *P* = 0.043). On the other hand, no significant differences were seen in the baseline characteristics age (64 ± 9 vs. 61 ± 11 years), BMI (27.3 ± 3.7 vs. 27.2 ± 3.8 kg/m^2^), BSA (2.0 ± 0.2 vs. 2.0 ± 0.2 m^2^) history of AF (19% vs. 10%) or prior ablation for AF (9% vs. 8%) (all *P* > 0.05). However, after stratification based on age (≤60y, 61-64y, 65-70y and ≥ 71y), there was more POAF in the oldest subgroup (≥71y)(POAF n = 15 vs. SR n = 7) compared to the youngest subgroup (≤60y) (POAF n = 14 vs. SR n = 20) (Table 1). Furthermore, there were no significant differences in preoperative drug use between POAF and non-POAF (Table S1).

**Table 1:** Baseline characteristics comparing patients with POAF to SR during hospitalization.

			POAF (n = 43)	SR (n = 40)	*P*-value	History of AF (n = 12)
*Demographics (n = 83)*						
	Age (years)		64 ± 9	61 ± 11	0.089	69 ± 7
		≤60	14 (33%)	20 (50%)	0.036	1 (8%)
		61–64	9 (21%)	3 (8%)		2 (17%)
		65–70	5 (12%)	10 (25%)		3 (25%)
		≥71	15 (35%)	7 (18%)		6 (50%)
	Female		10 (23%)	10 (25%)	0.853	3 (25%)
	BMI (kg/m^2^)		27.3 ± 3.7	27.2 ± 3.8	0.868	27.6 ± 2.5
	BSA (Dubois)		2.0 ± 0.2	2.0 ± 0.2	0.542	1.99 ± 0.1
	Logistic Euroscore		3.3 (2.2–4.7)	2.5 (1.5–4.4)	0.102	3.4 (2.6–12.8)
	Euroscore-II		1.2 (0.9–2.4)	1.2 (0.7–1.6)	0.382	1.7 (1.0–3.2)
	Additive Euroscore		4.0 (3.0–6.0)	3.5 (2.0–5.0)	0.110	4.5 (4.0–8.5)

*AF history*						
	Paroxysmal		8 (19%)	4 (10%)	0.354	12 (100%)
	Prior ablation		4 (9%)	3 (8%)	1.000	4 (33%)

*Comorbidities*						
	CHA_2_DS_2_-VASc		2 (1–3)	2 (1–3)	0.695	3 (2–5)
	COPD		5 (12%)	4 (10%)	1.000	1 (8%)
	OSAS		3 (7%)	7 (18%)	0.185	1 (8%)

*Echocardiographic measurements*						
	LA diameter (mm)		40 ± 6	40 ± 6	0.743	43 ± 6
	LA volume (ml)		82 ± 28	68 ± 21	0.039	74 ± 26
	LAVI (ml/m^2^)		42 ± 13	35 ± 11	0.043	37 ± 13
	LVEF (%)		59 ± 9	57 ± 8	0.437	58 ± 8

### 3.2 Procedural characteristics

All procedures were carried out at Maastricht University Medical Centre. Cardiac surgery consisted of surgery of the ascending aorta (with or without aortic valve repair n = 7, with aortic valve repair n = 6), isolated aortic valve surgery (repair n = 4, replacement n = 29) or stand-alone coronary artery bypass graft (CABG) (on-pump n = 28, off-pump n = 6). A combination of CABG and aortic valve surgery was performed in 9 patients.

Following the procedure, 43/83 (52%) patients developed POAF during hospitalization. The incidence of POAF was lower following isolated CABG surgery (32% POAF vs. 68% SR*, P* = 0.003), while no significant difference was seen for aortic valve (64% POAF vs. 36% SR, *P* = 0.080) or ascendens (86% POAF vs. 14% SR, *P* = 0.111) surgery. Of all patients undergoing CABG, only 6 patients received off-pump surgery (17% POAF vs. 83% SR, *P* = 0.101). No other surgery or perfusion-related aspects differed between the POAF and SR group (Table 2).

**Table 2:** Procedural characteristics of all patients undergoing cardiac surgery compared by rhythm outcome.

Characteristics			POAF (n = 43)	SR (n = 40)	*P*-value
*Type of surgery*					
	Aortic ascendens surgery (n = 7)				
		With aortic valve replacement or repair	6 (86%)	1 (14%)	0.111
		With aortic valve repair	5 (83%)	1 (17%)	0.203
	Aortic valve surgery (n = 33)				
		All	21 (64%)	12 (36%)	0.080
		Repair	2 (50%)	2 (50%)	1.000
		Replacement	19 (66%)	10 (34%)	0.067
	CABG (n = 34)				
		All	11 (32%)	23 (68%)	0.003
		Off pump	1 (17%)	5 (83%)	0.101
	Combination (n = 9)				
		CABG + aortic valve surgery	5 (56%)	4 (44%)	1.000

*Perfusion*					
	Lowest temperature (°C)		36 (34–36)	36 (36–36)	0.573
	Perfusion time (min)		87 (72–120)	84 (62–109)	0.252
	Aortic clamp time (min)		63 (48–84)	52 (41–74)	0.242

### 3.3 Epicardial adipose tissue measurements

**Fig. 2:**
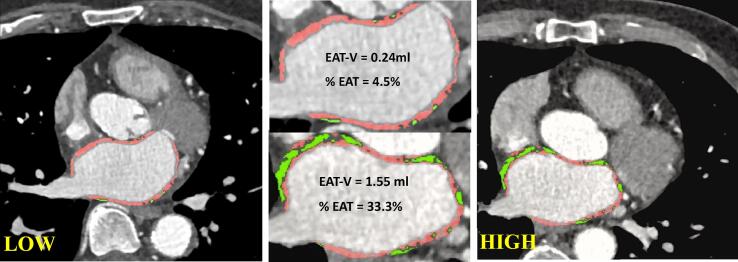
Left and upper middle: low EAT% in the LA wall (EAT-V = 0.22 ml, MYO-V = 4.63 ml, LA volume measured by TTE = 103 ml, EAT%=4.5 %). Right and upper middle: high EAT% in the LA wall (EAT-V = 1.55 ml, MYO-V = 3.11 ml, LA volume measured by TTE = 43 ml, EAT%=33.3 %). EAT-V = epicardial adipose tissue volume; MYO-V = myocardial volume; LA = left atrial; TTE = transthoracic echocardiogram.

EAT-V and MYO-V were calculated for all patients, with exclusion of the patients known with a history of AF (n = 12). An example of a patient with a relatively low and high EAT-V on CCTA is shown in Fig. 2. When comparing the volume of EAT between those with POAF and SR, no significant difference was seen (0.68 ml POAF vs. 0.67 ml SR, *P* = 0.431). Moreover, the percentage of EAT in the LA wall (19.15 ± 8.84 % vs. 19.34 ± 8.30 %) nor EAT-V indexed to BMI (2.67 [1.79–3.61] vs. 2.24 [1.36–3.79]), BSA (0.37 [0.27–0.49] vs. 0.33 [0.19–0.50]), LA volume (1.13 [0.83–1.56] vs. 0.93 [0.72–1.30]), LAVI (2.25 [1.51–3.04] vs. 1.83 [0.86–2.51]) and MYO-V (25.21 ± 14.46 vs. 25.26 ± 12.99) differed between both groups (all *P* > 0.05). Also EAT-V indexed to LA (ml) (EAT-LA) indexed to BSA, BMI, LAVI and MYO did not differ between groups either (Table 3). Furthermore, the percentages of EAT of the total LA wall, roof and non-roof did not statistically differ between POAF and SR (all *P* > 0.05), but patients with POAF did have a higher percentage of EAT in the roof compared to non-roof (23.28 ± 12.65 vs. 18.23 ± 8.91, *P* = 0.020) (Table 3, Table 4).

**Table 3:** EAT-V and EAT percentage in the LA wall, based on post-operative rhythm outcome. EAT-V were also indexed to BSA, BMI, LAVI and MYO-V.

Variables		POAF (n = 35)	SR (n = 36)	*P*-value
*EAT LA wall*				
	EAT-V (ml)	0.68 (0.51–1.04)	0.67 (0.41–0.97)	0.431
	MYO-V (ml)	2.87 (2.36–3.80)	2.88 (2.09–3.62)	0.265
	% EAT	19.15 ± 8.84	19.34 ± 8.30	0.929
	EAT-V (ml)/BMI (kg/m^2^)	2.67 (1.79–3.61)	2.24 (1.36–3.79)	0.577
	EAT-V/BSA (m^2^)	0.37 (0.27–0.49)	0.33 (0.19–0.50)	0.505
	EAT-V/LAVI (ml/m^2^)(%)	2.25 (1.51–3.04)	1.83 (0.86–2.51)	0.210
	EAT-V (ml)/MYO-V (ml)(%)	25.21 ± 14.46	25.26 ± 12.99	0.987
	EAT-V/LA (ml)(%) (=EAT-LA)	1.13 (0.83–1.56)	0.93 (0.72–1.30)	0.203
	EAT-LA/BMI (%)	3.84 (3.13–6.19)	3.39 (2.25–5.00)	0.431
	EAT-LA/BSA	0.57 (0.42–0.82)	0.49 (0.35–0.67)	0.272
	EAT-LA/MYO-V	0.36 (0.16–0.53)	0.32 (0.25–0.45)	0.942
	MYO-V/LAVI (%)	7.94 (6.39–11.08)	7.81 (5.66–10.71)	0.490

*EAT LA wall roof*				
	EAT-V (ml)	0.12 (0.06–0.21)	0.09 (0.06–0.16)	0.120
	MYO-V (ml)	0.50 (0.34–0.70)	0.46 (0.31–0.60)	0.227
	% EAT	23.28 ± 12.65	19.62 ± 10.88	0.196
	EAT-V roof/LA (%) (=EAT-roof-LA)	0.19 (0.08–0.36)	0.13 (0.08–0.21)	0.131
	(EAT-roof-LA)/BMI (%)	0.66 (0.32–1.29)	0.46 (0.36–0.71)	0.235
	(EAT-roof-LA)/BSA (%)	8.87 (4.02–17.47)	6.33 (4.77–10.25)	0.168
	(EAT-roof-LA)/MYO (%)	0.37 (0.24–0.73)	0.30 (0.16–0.43)	0.318

*EAT LA wall non -roof*				
	EAT-V (ml)	0.54 (0.34–0.87)	0.53 (0.32–0.85)	0.760
	MYO-V (ml)	2.46 (1.95–3.57)	2.33 (1.81–2.99)	0.204
	% EAT	18.23 ± 8.91	19.19 ± 8.51	0.524

**Table 4:** Comparison of the percentage EAT of the total LA wall, in the roof and non-roof for POAF and SR.

Groups	% EAT roof	% EAT non-roof	*P*-value
POAF (n = 35)	23.28 ± 12.65	18.23 ± 8.91	0.020
SR (n = 36)	19.62 ± 10.88	19.19 ± 8.51	0.753

Following univariate analyses, EAT-V variables were positively correlated with MYO-V and the percentage in the LA wall, but not with POAF. A weak positive trend was seen for age and EAT-V normalized to LA volume normalized to BSA, but this was not significant (r = 0.270, *P* = 0.053). After multivariable logistics regression analysis (with the exclusion of patients with pre-operative AF) using age, LAVI (ml/m^2^), the total percentage of EAT and the percentage of EAT in the roof, only age and LAVI were independent predictors for developing POAF (odd’s ratio (OR) = 1.076 [1.007–1.149], *P* = 0.030 and 1.056 [1.001–1.115], *P* = 0.047, respectively), while the percentages of total EAT and EAT in the roof were not (OR = 0.975 [0.883–1.077], *P* = 0.621 and 1.070 [0.990–1.156], *P* = 0.089, respectively) (Table S2).

## 4 Discussion

In this study we quantified EAT-V in the left atrial wall. We found that no local EAT-V variables were associated with POAF following cardiac surgery, while advanced age and LAVI were independent predictors of POAF.

### 4.1 Mechanisms underlying EAT and AF

Although previous studies have highlighted the role of EAT in AF, the exact mechanisms of the underlying pathophysiological process remain unclear [10]. In this process, two main potential arrhythmogenic mechanisms can be distinguished. First, EAT directly influences atrial electrophysiological properties by fatty infiltration into the contiguous epicardial layer of the myocardium [11], resulting in shortening of the atrial refractoriness, thereby favouring local conduction block and micro re-entry circuits [12], [13]. Secondly, paracrine secretion of factors such as adipokines can influence the direct adjacent myocardium, thereby leading to oxidative stress, autonomic- and diastolic dysfunction and adipocyte-related atrial gene expression [14], [15], [16]. Eventually, a combination of these factors can induce myocardial inflammation and fibrosis, resulting in slow but progressive structural remodelling and stabilization and perpetuation of AF [17]. On the contrary, Antonopoulos et al. reported a potential protective effect of atrial EAT in relation to oxidative stress [18]. During AF oxidative stress leads to an alteration in gene expression in EAT, such as the adipokine adiponectin. In the human heart, adiponectin in turn decreases myocardial oxidative stress via endocrine or paracrine effects, thereby representing a novel defence mechanism against myocardial oxidative stress. In addition to its role in inflammation and oxidative stress, EAT is involved in lipid and energy homeostasis by secreting free fatty acids [17]. Compared to other visceral depots, it has lower glucose utilization and also has brown fat properties that are hypothesized to protect the myocardium against hypothermia by producing free fatty acids [17]. Non-invasive imaging techniques can be used to measure adipose tissue as a surrogate marker of inflammation [19]. As the attenuation of inflamed adipocytes increases, inflamed cells can be distinguished from non-inflamed cells by CT-scan analyses based on the HU. Furthermore, PET-scan analysis could be used to provide real-time visualization of metabolic and inflammatory activity by the uptake of radioactive tracers within adipose tissue, thus providing more information on the metabolic characteristics of adipose tissue in a certain region of interest [22]. Unfortunately, these kinds of analyses have only been described on *peri*-coronary adipose tissue and not yet on fatty tissue on the atrial level. Subsequently, these analyses have not yet been validated for atrial adipose tissue and therefore not yet applicable in our population.

### 4.2 EAT and POAF

Since EAT is involved in the early phase of structural remodelling leading to AF, but has also shown to have cardioprotective effects, it is interesting to investigate the role of these early remodelling processes of EAT in the onset of POAF, especially given the transient occurrence of early POAF. In the postoperative setting, Opolski et al. [20] studied independent predictors of POAF after CABG in 108 patients. They found that LA EAT, measured by a comprehensive CCTA analysis using semi-automated software, is a significant predictor of POAF (OR: 1.21). It must be noted that 7% of the included study population had paroxysmal AF, while we excluded patients with diagnosed AF from the EAT analysis as it is known that EAT is associated with the occurrence and severity of AF by the release of adipokines leading to various alterations of the structural and functional properties of the atrial myocardium [17]. Furthermore, in the study by Drossos et al. a strong association between pericardial adipose tissue and POAF was reported following CABG [21]. However, the authors analysed a mixture of epicardial and pericardial fat and performed whole heart instead of selective atrial analysis. However, in the pathogenesis of AF, the arrhythmic effects of EAT seem local rather than systemic. Furthermore, our study used 2D source data for analysis, while the study of Drossos et al. performed their analysis based on a 3D reconstruction, which may have altered the morphological composition of EAT [21].

Kogo et al. analysed the association between EAT and POAF based on local EAT analysis, and did not find an association [22]. Also we did not find a significant correlation between EAT and POAF incidence. It seems that local EAT analysis does not allow to find an association between the quantification of EAT and the onset of POAF, while whole heart analysis of EAT does suggest that there is an association. As such, it might be that analysis of local EAT is not sensitive enough to find an association between EAT and the onset of POAF, but EAT analysis on the level of the whole heart is. On the other hand, it is known that in the acute postoperative phase, surgical induced factors (such as sympathetic activation, oxidative stress and inflammation) and hemodynamic and biochemical instability are known to elicit POAF also in patients with less severe AF substrates [4], [5]. As we performed our EAT analysis in a relatively low-risk population without a history of AF and a LAVI of 38 ± 12 ml/m^2^, the dominance of these acute factors might obscure the effects of a predisposing substrate in this low risk population.

### 4.3 Limitations

This study has some limitations. First, the retrospective character of the study combined with a small number of patients included in this analysis limits drawing valid conclusions. Patients with mitral valve disease were excluded since the underlying aetiology of AF is probably different as mitral valve disease causes significant left atrial (LA) dilatation as the result of mitral regurgitation [23]. However, despite this, predictors of POAF such as LA dimension and age were also found to be predictive for POAF in the present study. Secondly, while manual CT-scan analysis is considered a valid technique for measuring EAT and semi-automated quantification in custom made software allows for manual delineation of all regions of interest [9], no 'gold standard' overcoming human error in the analysis of EAT is yet available to compare our results with. Not only is manual delineation using semi-automated software time consuming (±45 min per scan), it can also be difficult to determine the exact anatomic borders of the ROI, such as the caudal and cranial part of the LA, as well as the entry points of the pulmonary veins into the LA. Finally, since both scan and CM injection parameters are patient specific and may differ between scanners, potential bias based on the heterogeneity in the attenuation of EAT as measured in HU cannot be excluded in this study. Although probably less important than LA EAT, as AF most often derives from the PV ostia and the posterior LA, right atrial EAT could not adequately be analysed in the present study due to the lack of contrast in the right atrium at the time of scanning.

## 5 Conclusion

This study found that advanced age and LAVI were independent predictors of POAF and that patients with POAF have significantly more EAT in the roof compared to non-roof. However, local EAT analysis was not associated with the occurrence of early POAF following cardiac surgery. This might suggest that early postoperative triggers dominate the ignition of early POAF, which potentially obscure the association of POAF with EAT, or that general rather than local effects of EAT play a role in the onset of POAF. Furthermore, perhaps not only the quantification of LA EAT is interesting to analyze, but also its metabolic and inflammatory properties. Therefore, it would be interesting if larger and prospective future studies combined PET-CT scan volume with inflammatory and/or metabolic activity analyses to compare not only the quantity of EAT between POAF and non-POAF, but also the metabolic and inflammatory characteristics of EAT, both systemic and local.

## Grant support

None.

## Declaration of Competing Interest

The authors report no relationships that could be construed as a conflict of interest.
