# Supplementary material for: Postoperative atrial fibrillation and atrial epicardial fat: Is there a link?
Source: Int J Cardiol Heart Vasc. 2022 Feb 21;39:100976. doi: 10.1016/j.ijcha.2022.100976 (PMC8984634; doi:10.1016/j.ijcha.2022.100976)
Supplement: Supplementary data 1 [file mmc1.docx]

**Postoperative Atrial Fibrillation and Atrial Epicardial Fat: is there a link?**

**Online Supplement**

**Table Legends**

**Table S1:** Data are presented as frequencies: n (%). AAD=antiarrhythmic drug; HCN=hyperpolarization-activated cyclic nucleotide-gated.

**Table S2:** Data are presented as odd’s ratio [95% confidence interval], *p*-value. EAT=epicardial adipose tissue; LAVI=left atrial volume index; POAF=postoperative atrial fibrillation.

**Tables**

**Table S1**: Drug use in patients with POAF, SR and AF.

|  | POAF (n=43) | SR (n=40) | *P*-value | History of AF (n=12) |
| --- | --- | --- | --- | --- |
| Ace-inhibitor | 11 (31%) | 7 (19%) | 0.246 | 3 (25%) |
| Antibiotic | 5 (14%) | 2 (6%) | 0.260 | 2 (17%) |
| Anti-diabetic | 6 (17%) | 2 (6%) | 0.151 | 3 (25%) |
| Antidepressant | 1 (3%) | 6 (17%) | 0.107 | 2 (17%) |
| Angiotensin-receptor blocker | 7 (20%) | 8 (22%) | 0.819 | 5 (42%) |
| Benzodiazepine | 2 (6%) | 7 (19%) | 0.151 | 1 (8.3%) |
| Beta-blocker | 20 (57%) | 17 (47%) | 0.403 | 5 (42%) |
| Calcium antagonist | 12 (34%) | 8 (22%) | 0.259 | 5 (42%) |
| Diuretic | 8 (23%) | 8 (22%) | 0.949 | 8 (67%) |
| HCN channel blocker | 0 (0%) | 2 (6%( | 0.493 | 0 (0%) |
| Lowering cholesterol | 19 (54%) | 23 (64%) | 0.411 | 11 (92%) |
| Nitrate | 4 (11%) | 8 (22%) | 0.225 | 2 (17%) |
| Oral-anticoagulation | 2 (6%) | 1 (3%) | 0.614 | 5 (42%) |
| Proton pump inhibitor | 12 (34%) | 17 (47%) | 0.268 | 6 (50%) |

**Table S2:** Multivariable logistic regression analysis.

| Clinical variables | POAF |
| --- | --- |
| Age (years) | 1.076 [1.007 – 1.149], P = 0.030 |
| LAVI (ml/m^2^) | 1.056 [1.001 – 1.115], P = 0.047 |
| Total LA EAT (%) | 0.975 [0.883 – 1.077], P = 0.621 |
| EAT LA roof (%) | 1.070 [0.990 – 1.156], P = 0.089 |
